# Enrichment of Cryoconite Hole Anaerobes: Implications for the Subglacial Microbiome

**DOI:** 10.1007/s00248-016-0886-6

**Published:** 2016-11-07

**Authors:** Marek K. Zdanowski, Albert Bogdanowicz, Jan Gawor, Robert Gromadka, Dorota Wolicka, Jakub Grzesiak

**Affiliations:** 1 Department of Antarctic Biology, Institute of Biochemistry and Biophysics, Polish Academy of Sciences, Pawińskiego 5a, 02-106 Warsaw, Poland; 2 Laboratory of DNA Sequencing and Oligonucleotide Synthesis, Institute of Biochemistry and Biophysics, Polish Academy of Sciences, Pawińskiego 5a, 02-106 Warsaw, Poland; 3 Institute of Geochemistry, Mineralogy and Petrology, Faculty of Geology, Warsaw University, Warsaw, Poland

**Keywords:** Bacteria, Glacier, Psychrophiles, *Firmicutes*, Methanogenesis

## Abstract

Glaciers have recently been recognized as ecosystems comprised of several distinct habitats: a sunlit and oxygenated glacial surface, glacial ice, and a dark, mostly anoxic glacial bed. Surface meltwaters annually flood the subglacial sediments by means of drainage channels. Glacial surfaces host aquatic microhabitats called cryoconite holes, regarded as “hot spots” of microbial abundance and activity, largely contributing to the meltwaters’ bacterial diversity. This study presents an investigation of cryoconite hole anaerobes and discusses their possible impact on subglacial microbial communities, combining 16S rRNA gene fragment amplicon sequencing and the traditional enrichment culture technique. Cryoconite hole sediment harbored bacteria belonging mainly to the *Proteobacteria* (21%), *Bacteroidetes* (16%), *Actinobacteria* (14%), and *Planctomycetes* (6%) phyla. An 8-week incubation of those sediments in Postgate C medium for sulfate reducers in airtight bottles, emulating subglacial conditions, eliminated a great majority of dominant taxa, leading to enrichment of the *Firmicutes* (62%), *Proteobacteria* (14%), and *Bacteroidetes* (13%), which consisted of anaerobic genera like *Clostridium*, *Psychrosinus*, *Paludibacter*, and *Acetobacterium*. Enrichment of *Pseudomonas* spp. also occurred, suggesting it played a role as a dominant oxygen scavenger, providing a possible scenario for anaerobic niche establishment in subglacial habitats. To our knowledge, this is the first paper to provide insight into the diversity of the anaerobic part of the cryoconite hole microbial community and its potential to contribute to matter turnover in anoxic, subglacial sites.

## Introduction

Glaciers and ice sheets are known as the largest freshwater reservoirs on Earth, comprising approx. 70% of its resource [20, 27]. Only recently have those systems been recognized as biomes, hosting mostly microbial life and microbe-mediated processes [1]. Within the boundaries of a glacial ecosystem, two environments were found to be of major ecological importance and gained much scientific interest: (1) the subglacial environment, consisting of a boundary between basal ice and bedrock, where a thin layer of water is produced by friction and geothermal heat; (2) the supraglacial environment, comprising of snow, ice, and related features like cryoconite holes [13]. Cryoconite holes are considered “hot spots” of microbial life on glacial surfaces as they harbor elevated amounts of cells compared to surrounding ice [12]. They form as water-filled depressions, when dark, wind-derived debris melts into the ice. Those holes receive abundant sunlight and allochthonous inputs of biogenic substances. Intense ablation causes the holes to merge by melting the surrounding ice. A drainage system develops, delivering supraglacial meltwaters via crevasses and moulins to the glacial bed [9]. Supraglacial meltwaters have low conductivity, meaning they do not carry many ions (inorganic salts) [4]. Yet, they contain labile organic carbon, freshly produced via photosynthesis by phototrophic communities or direct photolysis of recalcitrant, aromatic compounds of allochthonous origin as well as viable microbes, including bacteria, archaea, and fungi [5]. Upon reaching the subglacial zone, those meltwaters experience chemical enrichment in many minor and trace elements over relatively short distances [4]. Subglacial sediments get aerated in proximity to active drainage channels but become anoxic during winter, when basal melting dominates the supply of water [30].

Since the delivery of supraglacial microbes to subglacial environments by the meltwater runoff is highly efficient [14], we wanted to know what part of the cryoconite bacterial community can thrive under subglacial conditions and what could be their role in contributing to anaerobic matter turnover. Research on subglacial discharge indicates that in the majority of glaciers, it contains considerable amounts of sulfates [17] and simple organic acids such as labile carbon compounds [19]. Therefore, we chose the Postgate C medium for sulfate reducers and the airtight microcosm method to emulate subglacial conditions and enrich cryoconite hole anaerobes. A phylogenetic diversity comparison of the enriched and native bacterial communities was made by means of next generation sequencing (Illumina MiSeq) of 16S ribosomal RNA (rRNA) gene amplicons. We hypothesize that anaerobic enrichment will cause emergence of a narrow group of bacterial taxa, most probably endospore formers of the phylum *Firmicutes*, moderately abundant in open-air cryoconite holes. This study, combining a 16S rRNA gene fragment amplicon sequencing and the traditional enrichment culture technique, provides valuable insight into the ecology of glaciers, especially in terms of cryoconite hole anaerobes, which have not been investigated to date and discusses their potential role as contributors to subglacial microbial communities.

## Materials and Methods

### Sites and Sampling

Ecology Glacier is situated at the western shore of Admiralty Bay, on King George Island, South Shetland Archipelago, Antarctica. In December 2011/January 2012 when sampling took place, there were numerous cryoconite holes on Ecology Glaciers surface. Cryoconite sediment material was collected aseptically at four sites on the surface of Ecology Glacier into chemically clean and sterile containers along a transect running from the glacier terminus to the snow line at the top of the ablation zone (more details, including physicochemical and microbiological measurements [12]). Cryoconite holes were drained of water and sediment with a 160-mL sterile plastic syringe, and the material was transported in 500-mL sterile bottles to a field laboratory and processed within 2 h. Five cryoconite holes per site were drained and pooled.

### Media and Inoculations

One hundred-milliliter screw cap bottles (Simax) were filled to a volume of 80 mL with Postgate C medium, containing Na-lactate (6.0 g/L), Na_2_SO_4_ (4.5 g/L), NH_4_Cl (1.0 g/L), yeast extract (1.0 g/L), KH_2_PO_4_ (0.5 g/L), Na-citrate·2H_2_O (0.3 g/L), CaCl_2_·6H_2_O (0.06 g/L), MgSO_4_·7H_2_O (0.06 g/L), and FeSO_4_·7H_2_O (0.04 g/L), heat sterilized, and cooled to approx. 4 °C. Three bottles per site were inoculated with 20 mL of cryoconite sediment-water mix and sealed. Incubation was carried out at 4 °C for 10 weeks. Sulfide precipitation was indicated by Wolicka et al. [34].

### Microscopy

Fifty microliter of each of the enrichment cultures after the 10-week incubation time was placed on a basic glass slide and allowed to air dry. The dried suspensions on glass slides were fixed in a laboratory burner flame, followed by a Gram-staining procedure using Biomerieux reagents according to manufacturer’s protocol. A duplicate set of slides was subjected to an endospore-staining procedure as described by Bartholomew and Mittwer [3].

### DNA Extraction

One milliliter of cryoconite sediment–water mix was placed in a 2-mL sterile plastic Eppendorf-type tube and centrifuged at 9000 rpm for 3 min in an MPW-52 microcentrifuge to separate the sediment from the water. Total DNA was extracted with the use of a PowerSoil® DNA Isolation Kit (MoBio, Carlsbad, CA, USA) in accordance with the manufacturer’s protocol. The same procedure was done with the post-culture deposits.

### 16S rRNA Gene Amplicon Library Preparation and Illumina Sequencing

Pooled samples from several cryoconite holes and their respective enrichment cultures were examined. Phylogenetic study was performed by sequencing and analysis of prokaryotic 16S ribosomal RNA gene. A fragment of the16S rRNA gene containing the V3 and V4 variable regions was amplified using gene-specific primers: 16S_V3-F and 16S_V4-R positions 341-357F and 785-805R, respectively, according to *Escherichia coli* 16S rRNA gene reference sequence [15]. Illumina Nextera XT overhang adapter nucleotide sequences were included in addition to the 16S rRNA gene-specific sequences, which allowed sample indexing and pooling. Each PCR amplification was done in triplicate using KAPA HiFi PCR using a final volume of 20 μL per reaction according to the manufacturer’s instructions. Obtained PCR products were pooled in equimolar ratio and indexed using Nextera XT barcodes (Illumina, San Diego, USA). Amplicon libraries were pooled and sequenced on Illumina MiSeq instrument (Illumina, San Diego, USA) in the DNA Sequencing and Oligonucleotide Synthesis Laboratory (Institute of Biochemistry and Biophysics, Polish Academy of Sciences). Sequencing was done in paired-end mode (2 × 300 bp) with the use of a v3 (600 cycles) chemistry cartridge which allowed generation of long paired reads fully covering 16S V3–V4 amplicons.

### Sequence Processing and Analysis

Raw sequencing data were preliminarily cleaned with FASTX-Toolkit (http://hannonlab.cshl.edu/fastx_toolkit/) based on FastQC (http://www.bioinformatics.babraham.ac.uk/projects/fastqc/) quality assessment. Further analysis was done with Mothur [25], following a standard operating procedure optimized for Illumina MiSeq long paired-end reads, as described on the Mothur’s website, with the Silva 16S database [22] as reference. An additional attempt at classifying sequences was made using the classifier from the Ribosomal Database Project (RDP) Pipeline [33]. Sequences obtained from native and enriched samples were deposited in the NCBI Sequence Read Archive (SRA) under the accession numbers SRR3476896 and SRR3476921, respectively.

## Results

All bottles containing Postgates C medium for sulfate reducers inoculated with cryoconite sediments displayed formation of characteristic black precipitate after the 8-week incubation period. Pooled samples from several cryoconite holes and their respective enrichment cultures were examined. The V3 and V4 regions of the bacterial 16S rRNA gene were amplified and sequenced using MiSeq sequencer. Illumina sequencing yielded 28,857 sequences from native cryoconite hole samples; 278,994 sequences were obtained from enrichment cultures in Postgates medium C for sulfate reducers.

### Native Cryoconite Hole Bacterial Community Structure

**Fig. 1:**
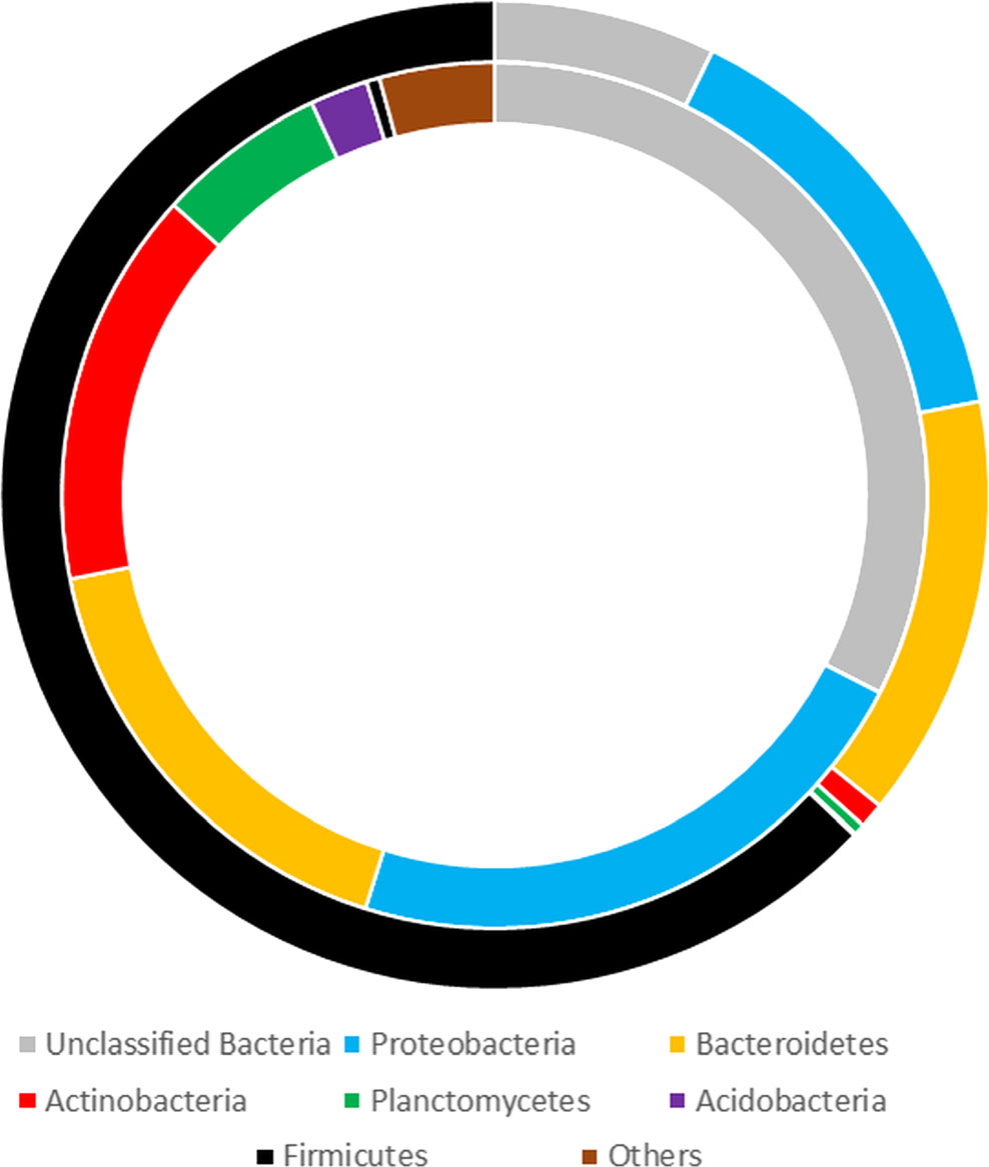
Taxonomic bacterial community structure based on sequence abundance in native cryoconite samples (*inner circle*) and in the enrichment culture following 8 weeks of incubation (*outer circle*)

**Fig. 2:**
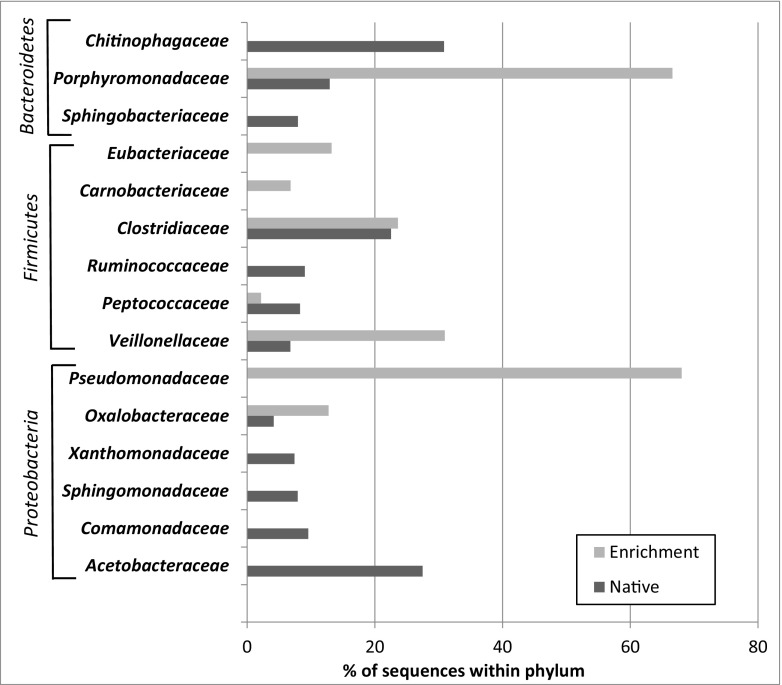
Percentage of sequences identified on family level within three major enriched phyla: *Firmicutes*, *Bacteroidetes*, and *Proteobacteria*

Based on sequence abundance, the bulk of bacterial community in native cryoconite hole sediment consisted of *Proteobacteria* (21%), *Bacteroidetes* (16%), *Actinobacteria* (14%), and *Planctomycetes* (6%), although over 30% of the sequences within the bacterial domain remained unclassified. *Firmicutes* were a marginal group within the native samples (0.47% of sequences) (Fig. 1). Within the *Proteobacteria* phylum, sequences of the families *Acetobacteraceae* (27.5%), *Comamonadaceae* (9.5%), *Sphingomonadaceae* (7.9%), and *Xanthomonadaceae* (7.4%) were the most abundant in native samples. Forty-two percent of the native sample sequences classified as *Bacteroidetes* were unassigned by the RDP classifier pipeline at a family level. Thirty percent of the sequences within this phylum belonged to the *Chitinophagaceae* family, 13% to *Porphyromonadaceae*, and 7.9% to *Sphingobacteriaceae*. Nearly half of *Firmicutes* sequences were not classifiable at the family level, 22% accounted for *Clostridiaceae*, 9% for *Ruminococcaceae*, 8.2% *Peptococcaceae*, and 6% for *Veillonellaceae* (Fig. 2).

### Bacterial Community Structure After Enrichment Procedure

**Fig. 3:**
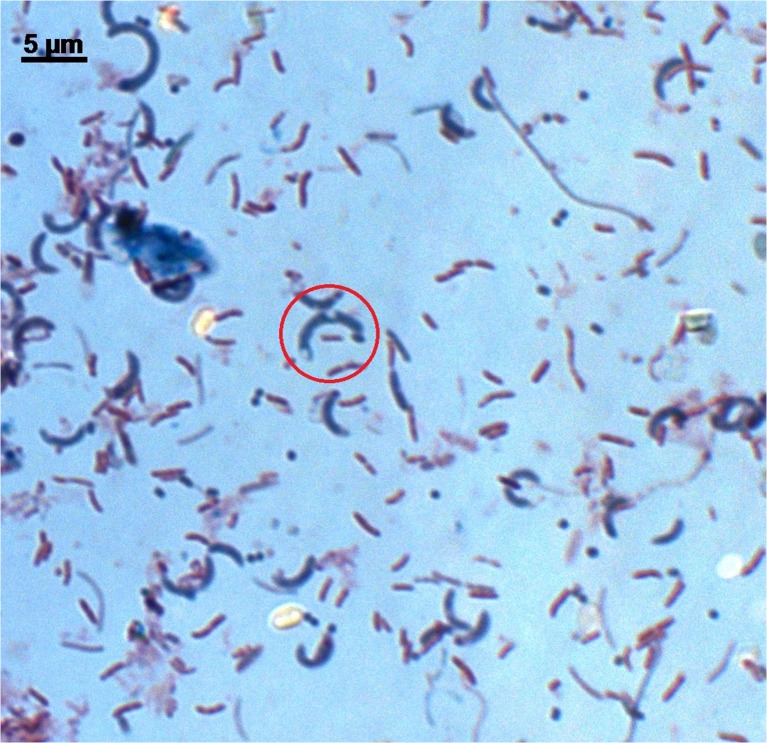
Photomicrograph of Gram-stained enrichment culture, showing several cell morphotypes, including large, curved, endospore-forming (*red circle*) cells, characteristic for the *Clostridia* class within *Firmicutes* phylum

The enrichment procedure has drastically changed the bacterial community structure within the sediment, promoting cell proliferation of the phylum *Firmicutes*, which increased its sequence contribution to >62%. *Proteobacteria* and *Bacteroidetes* representatives’ sequences contributed 14 and 13%, respectively. *Actinobacteria* and *Planctomyces* sequences were reduced in numbers to less than 1% (Fig. 1). The community structure within the three enriched types (*Proteobacteria*, *Firmicutes*, and *Bacteroidetes*) also underwent considerable changes. *Proteobacteria* sequences in enrichment cultures were dominated by members of the family *Pseudomonadaceae*, which were not detected in native samples. *Oxalobacteraceae* sequences also contributed to the enriched samples by 12%. Based on sequence abundance in enrichment cultures, *Veillonellaceae* became the dominant group within the *Firmicutes* phylum, with 30% of sequences; *Clostridiaceae* members accounted for 23 % of sequences and the *Eubacteriaceae* 13%. *Carnobacteriaceae*, undetected in native samples, scored 6.8% within the *Firmicutes* phylum. In the enrichment culture, the *Bacteroidetes* phylum was dominated by members of the *Porphyromonadaceae* family with 66% of sequences (Fig. 2). At the identified genus level, the groups that were considerably enriched by the procedure used were the following: *Psychrosinus*, *Clostridium*, *Paludibacter*, *Acetobacterium*, *Pseudomonas*, *Carnobacterium*, and *Desulfosporosinus* (Table 1). Curved, endospore-forming cells that stained Gram variable were the most characteristic features seen in post-culture deposits (Fig. 3).

**Table 1:** Percentage of sequences of genera considerably enriched in the Postgate C medium

Phylum	Family	Genus	Native samples (% of total sequences)	Enrichment (% of total sequences)
*Proteobacteria*	*Pseudomonadaceae*	*Pseudomonas*	ND	7.6
*Firmicutes*	*Veillonellaceae*	*Psychrosinus*	0.013	11.25
*Firmicutes*	*Clostridiaceae*	*Clostridium*	0.05	10.53
*Firmicutes*	*Eubacteriaceae*	*Acetobacterium*	0.007	7.8
*Firmicutes*	*Peptococcaceae*	*Desulfosporosinus*	0.003	1.34
*Firmicutes*	*Clostridiaceae*	*Sedimentibacter*	0.003	0.83
*Firmicutes*	*Carnobacteriaceae*	*Carnobacterium*	ND	3.4
*Bacteroidetes*	*Porphyromonadaceae*	*Paludibacter*	1.9	9

*ND* not detected

## Discussion

Cryoconite holes can generally be regarded as aerobic environments. Dissolved oxygen concentrations measured in situ in cryoconite hole water at Ecology Glacier were in the range of 8 to 11 mg/L at 0 °C which equates to 57 to 78% saturation [16]. Bagshaw et al. [2] demonstrated that even in ice-lidded cryoconite holes, there is abundant dissolved oxygen due to high O_2_ solubility in low temperatures and also, the release of air bubbles trapped in glacial ice while melting. Yet, viable strict anaerobes have been detected and enriched from the sediment in this study, although they constituted a small fraction of the microbial community (as indicated by low sequence numbers). In several other environments, such phenomena have been explained by anoxic microhabitats, created by biofilm formation and oxygen depletion by aerobes [8]. Indeed, denitrification processes were detected in cryoconite granules that were less than 2 mm in diameter, where *Polaromonas* cells have been proposed as O_2_ scavengers and extracellular polymer producers, causing clumping of the sediment [26]. *Polaromonas* spp. have also been described as a member of bacterial communities on Ecology Glacier surface [11], likely contributing with other O_2_ consumers and biofilm formers to anaerobic microniche establishment.

A great majority of dominant aerobic taxa like *Actinobacteria* or the *Proteobacteria* families of *Acetobacteraceae* and *Comamonadaceae* have been eliminated from the bacterial community during the enrichment process, despite not using reducing agents like cysteine or thioglycollate or purging the O_2_ with non-reactive gases. Cells of the genus *Pseudomonas* that proliferated under enrichment conditions may have depleted the available oxygen in early stages of the procedure; thus, aiding anaerobes in establishing an abundant community, *Pseudomonas* and other *Gammaproteobacteria* are known as versatile opportunists, dwelling in habitats, where conditions frequently change [18]. One such example of cooperation between *Desulfovibrio oxyclinae* (a sulfate-reducing bacterium) and *Marinobacter* sp. (class *Gammaproteobacteria*) has been described under exposure to increasing oxygen concentrations [28]. This phenomenon points towards a possible scenario for anaerobic niche formation in subglacial sediments, especially after supraglacial meltwater delivery channel closure.

The enrichment procedure did not only recover endospore formers, but also strictly anaerobic non-endospore-forming genera. Based on sequence abundance, members of the genus *Psychrosinus* were the most enriched bacteria in this study. Although belonging to the phylum *Firmicutes*, those bacteria do not form endospores. They were primarily found in Lake Fryxell (Antarctica), where they dwelled in the sulfidic monimolimnion and fermenting lactic acid [23], which was the main carbon source in the enrichment medium. Non-endospore-forming members of this phylum like *Acetobacterium* and *Carnobacterium* that have been enriched are also present in anoxic parts of Antarctic lakes [10, 24], which would suggest, that those genera are readily dispersed in the Antarctic region, and develop in abundance when the right conditions are provided. Enriched endospore formers, like *Clostridium* spp., have also been detected in anoxic lake sediments in Antarctica. They were involved in proteolysis in nutrient-rich environments [6], very unlike the oligotrophic cryoconite sediment [12]. An interesting case presents the detection of *Paludibacter* (*Bacteroidetes*). With only two species described, both isolated from paddy rice fields, not much is known about their ecology [21, 31]. None of the described species displayed psychrophilic growth characteristics. Sequences closely related to members of the *Desulfosporosinus* genus have been identified in both natural and enriched cryoconite bacterial communities and are proposed to be responsible for sulfate reduction and consequent blackening of the medium.

Detection of a great diversity of viable strict anaerobes in cryoconite sediments and their considerable enrichment in the Postgate C medium presents profound implications for subglacial microbial communities, especially when methanogenesis is concerned as it has been widely recognized to occur under glaciers and ice-sheets worldwide [7, 32]. Although methanogens were not detected in this study, bacteria of the genus *Clostridium* and *Acetobacterium* have been frequently observed forming syntrophic associations with methanogenic *Archaea*, fermenting carbon compounds of varying complexity and producing acetate, CO_2_, and hydrogen—substances pivotal for microbial CH_4_ production [29]. Acetate production has also been observed with *Paludibacter* and *Psychrosinus* [21, 23, 31], suggesting they also may play a role in aiding methane production in subglacial sediments, although their direct link to methanogens has never been observed, posing opportunities for further research. Evidence for efficient acetate formation processes in those habitats was recently discovered by indicating acetate as the major organic acid in subglacial outflows [19].

## Conclusions

Based on sequence abundance, anaerobic bacteria residing in cryoconite holes constituted a small percentage of the whole bacterial community, indicating that the melt holes are not favorable environments for strict anaerobes. The applied enrichment procedure caused several anaerobic genera to proliferate, many of them non-endospore forming, like *Psychrosinus*, *Acetobacterium*, and *Paludibacter* proving that not only endospore formers can withstand the aerial transfer, deposition on a glacial surface, and the cryoconite hole formation process. Furthermore, they thrived under simulated subglacial conditions implying the possibility of cryoconite hole microbes to annually add to the taxonomic, eco-physiologic, and genetic diversity of subglacial anaerobic communities. Traits generally attributed to genera enriched in this study, like acetate and hydrogen production, aid the establishment of methanogen populations and the efficiency of CH_4_ production, being of special scientific concern regarding glacier-hosted microbial activity. This study contributes to the scarce knowledge of the largest freshwater reservoirs on Earth, namely glaciers, by providing information on the overlooked anaerobes of supraglacial aquatic microhabitats (cryoconite holes) and linking them with the subglacial environment.
